# Comparative transcriptomics in Syllidae (Annelida) indicates that posterior regeneration and regular growth are comparable, while anterior regeneration is a distinct process

**DOI:** 10.1186/s12864-019-6223-y

**Published:** 2019-11-14

**Authors:** Rannyele Passos Ribeiro, Guillermo Ponz-Segrelles, Christoph Bleidorn, Maria Teresa Aguado

**Affiliations:** 1 Departamento de Biología, Facultad de Ciencias, Universidad Autónoma de Madrid, Cantoblanco, 28049 Madrid, Spain; 2 Animal Evolution & Biodiversity, Georg-August-Universität Göttingen, 37073 Göttingen, Germany; 3 German Centre for Integrative Biodiversity Research (iDiv) Halle-Jena-Leipzig, 04103 Leipzig, Germany; 4 Centro de Investigación en Biodiversidad y Cambio Global (CIBC-UAM), Universidad Autónoma de Madrid, Madrid, 28049 España

**Keywords:** Regeneration, Annelida, Syllidae, RNA-seq, Transcriptome, *Hox* genes, β*-*Catenin, JNK, PL10

## Abstract

**Background:**

Annelids exhibit remarkable postembryonic developmental abilities. Most annelids grow during their whole life by adding segments through the action of a segment addition zone (SAZ) located in front of the pygidium. In addition, they show an outstanding ability to regenerate their bodies. Experimental evidence and field observations show that many annelids are able to regenerate their posterior bodies, while anterior regeneration is often limited or absent. Syllidae, for instance, usually show high abilities of posterior regeneration, although anterior regeneration varies across species. Some syllids are able to partially restore the anterior end, while others regenerate all lost anterior body after bisection. Here, we used comparative transcriptomics to detect changes in the gene expression profiles during anterior regeneration, posterior regeneration and regular growth of two syllid species: *Sphaerosyllis hystrix* and *Syllis gracilis*; which exhibit limited and complete anterior regeneration, respectively.

**Results:**

We detected a high number of genes with differential expression: 4771 genes in *S. hystrix* (limited anterior regeneration) and 1997 genes in *S. gracilis* (complete anterior regeneration). For both species, the comparative transcriptomic analysis showed that gene expression during posterior regeneration and regular growth was very similar, whereas anterior regeneration was characterized by up-regulation of several genes. Among the up-regulated genes, we identified putative homologs of regeneration-related genes associated to cellular proliferation, nervous system development, establishment of body axis, and stem-cellness; such as *rup* and *JNK* (in *S. hystrix*); and *glutamine synthetase*, *elav*, *slit, Hox* genes, β-*catenin* and *PL10* (in *S. gracilis*).

**Conclusions:**

Posterior regeneration and regular growth show no significant differences in gene expression in the herein investigated syllids. However, anterior regeneration is associated with a clear change in terms of gene expression in both species. Our comparative transcriptomic analysis was able to detect differential expression of some regeneration-related genes, suggesting that syllids share some features of the regenerative mechanisms already known for other annelids and invertebrates.

## Background

Growth and regeneration are remarkable developmental abilities of annelids. Most annelids grow during their entire life by addition of segments from a segment addition zone (SAZ) located between the pygidium and the last segment [1–8]. Moreover, nearly all annelid species are able to completely restore the posterior body, while only some groups are able to regenerate the anterior body [6–10]. Whereas several studies describing the process of (anterior and posterior) regeneration are available, the molecular background of this ability remains largely unknown in annelids [6–8, 10].

Studies including molecular data during regeneration in annelids have been published for the clitellates *Enchytraeus japonensis* Nakamura, 1993 [11–15] and *Pristina leidyi* Smith, 1896 [1, 16–20]; and the non-clitellates *Alitta virens* Sars, 1835 [21–24], *Capitella teleta* Blake, Grassle and Eckelbarger, 2009 [25–29], and *Platynereis dumerilli* (Audouin and Milne Edwards, 1833) [3, 30–35]. All those species regenerate the posterior body, but only *E. japonensis* and *P. leidyi* exhibit anterior regeneration [1, 11–15, 17–20]. Studies on anterior regeneration in non-clitellates have been limited to morphological approaches so far (e.g. [36–44]). Interestingly, some genes that are expressed in the SAZ during regular growth/development have been detected in different stages of posterior regeneration in annelids, for example, *Hox* genes [21–23, 27, 45], β-*catenin* [17], and genes of the germline multipotency program such as *piwi*, *vasa*, *nanos*, and *PL10* [27, 46–48].

Within Annelida, Syllidae are known to completely regenerate their tails [8, 49]. However, when dealing with anterior regeneration, many species can only regrow the prostomium and few segments, e.g. *Eusyllis blomstrandi* Malmgren, 1867 [49–51]; while others additionally regenerate all missing segments and also a characteristic differentiation of the digestive tube called proventricle (e.g. *Syllis gracilis* Grube, 1840 [37, 52–55]). Interestingly, the molecular background of regeneration in syllids has not been explored.

We used RNA-seq to generate gene expression profiles of the anterior and posterior regeneration processes, as well as the regular posterior growth of two species of syllids: *Sphaerosyllis hystrix* Claparède, 1863 [56] (Exogoninae)*,* and *Syllis gracilis* (Syllinae). Our aim was to analyse the changes in gene expression during the first stages of posttraumatic anterior regeneration (AR) and posterior regeneration (PR) by comparing them with the non-regenerating condition (NR) (i.e. intact individuals in regular posterior growth), and between themselves (AR and PR). Additionally, selected genes previously shown to be (highly) expressed during regeneration in other annelids and other invertebrates have been investigated. Finally, we also documented the morphological changes during anterior and posterior regeneration in both species, and identified regeneration-related genes that could be of interest for future studies in syllid regeneration.

## Results

### Illumina NGS and assembly

**Fig. 1:**
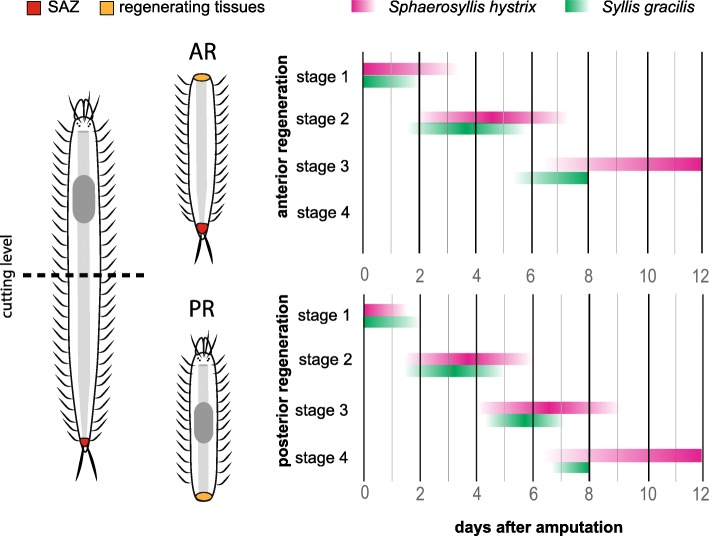
Regeneration timeline of the specimens sequenced for transcriptomic data. Bisection was performed in the midbody site and the amputees were fixed for sequencing in the first stages of regeneration: stage 1 (healing), stage 2 (early blastema development), stage 3 (late blastema development), and stage 4 (patterning/cap regeneration). Anterior regeneration sequencing cover stages 1–3; posterior regeneration covers all the stages. Time-scale of experimentation: 12 days for *Sphaerosyllis hystrix* and 8 days for *Syllis gracilis* (see Methods)

**Fig. 2:**
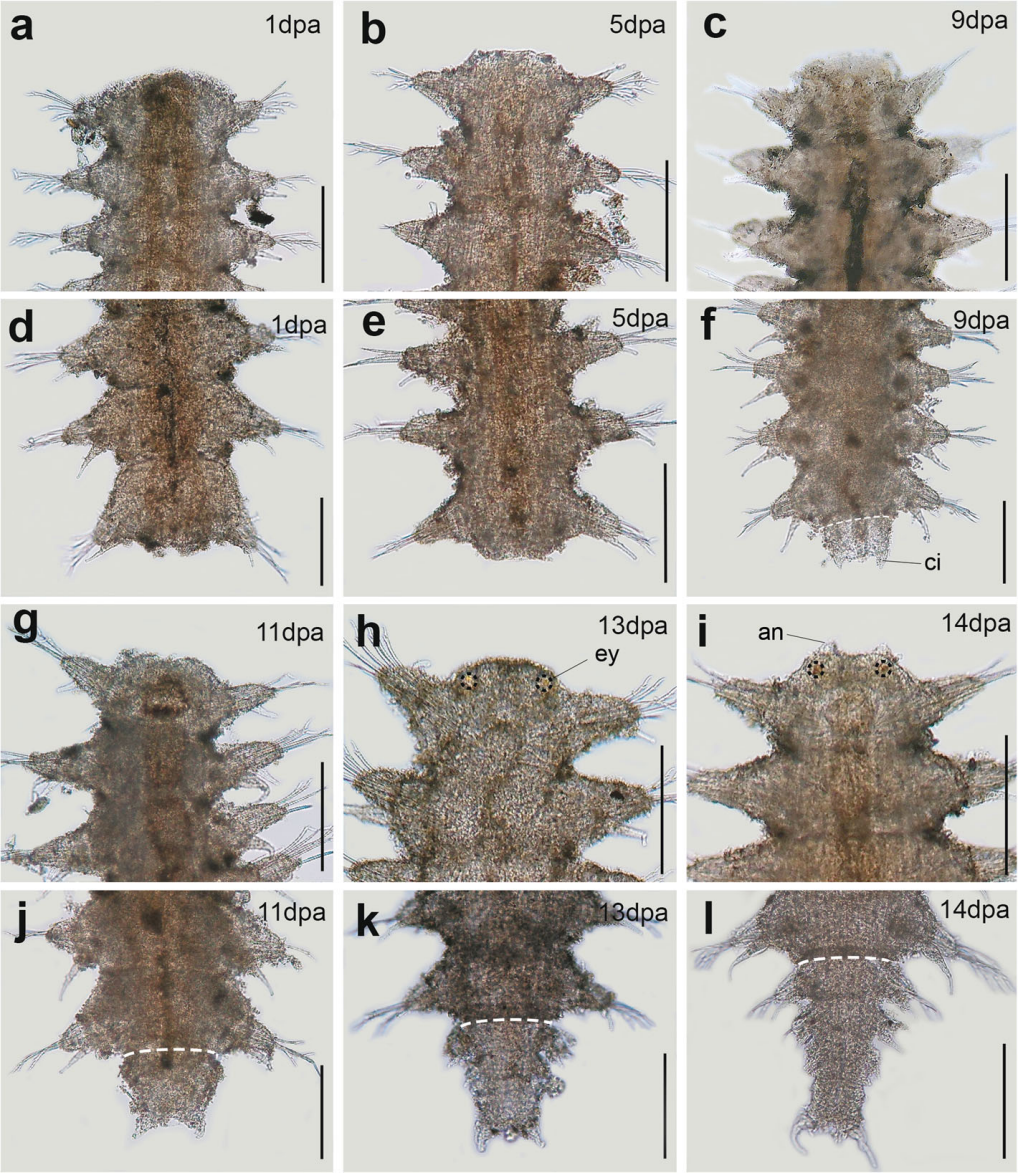
Light microscopy pictures of the regenerating *Sphaerosyllis hystrix*. **a**, **b**, **c**, **g**, **h**, **i** anterior regeneration. **d**, **e**, **f**, **j**, **k**, **l** posterior regeneration. Amputation was performed in the midbody region and the regenerating animals were observed for 14 days post amputation (dpa). Immediately after body bisection, the wound is closed by invagination through muscle contraction. Anterior regeneration starts by wound healing (1–3 dpa) and the formation of a small blastema (**a**). The anterior blastema is formed after 4–6 dpa and no differentiated organ is regenerated until 12 dpa (**b**, **c**, **g**). An incomplete prostomium (head) appeared after 13 dpa, bearing eyes (**h**), and a pair of minute antennae in 14 dpa (**i**). Posterior regeneration proceeds more quickly: healing occurred in 2 dpa, the blastema developed from 2 to 4 dpa, and a pygidium with a pair of cirri was first seen after 9 dpa (**d**, **e**, **f**). From 10 to 14 dpa, amputees had regrown new pygidia and a maximum of four posterior segments (**j**–**l**). All pictures are in dorsal view. Scale bar 0.2 mm. White dashed lines show amputation level. Black dashed lines show the regenerated eyes. Abs: an, antenna; ey, eye

**Fig. 3:**
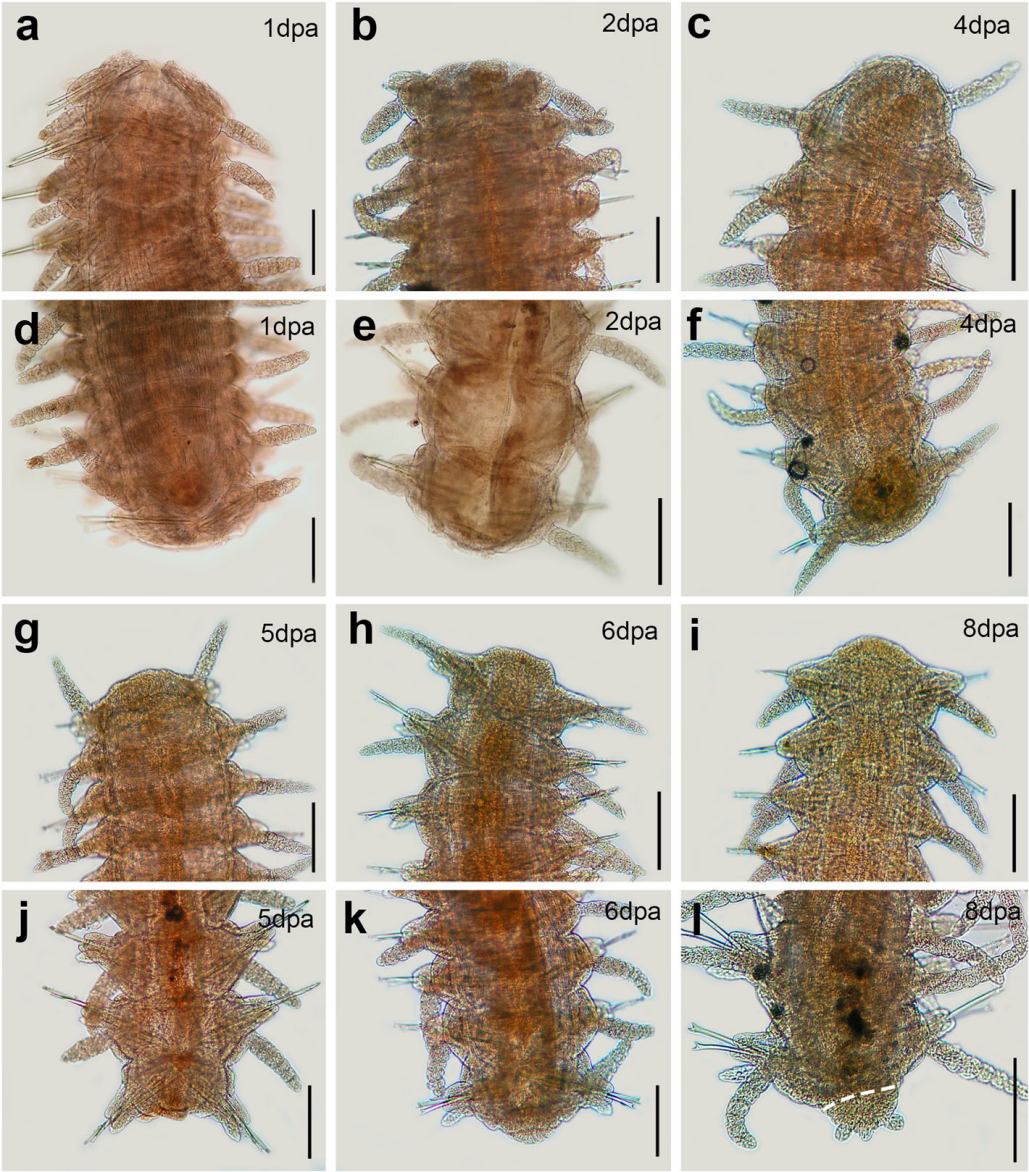
Light microscopy pictures of the regenerating *Syllis gracilis*. **a**, **b**, **c**, **g**, **h**, **i** anterior regeneration. **d**, **e**, **f**, **j**, **k**, **l** posterior regeneration. Anterior and posterior regeneration of *S. gracilis* were observed during 8 dpa. The wound is completely healed after 2 dpa and a blastema develops during the following days in both anterior and posterior regeneration. After 8dpa, the blastema was still elongating during anterior regeneration (**a**–**c**, **g**–**i**). Regarding posterior regeneration, the blastema differentiated between 4 and 7 dpa; after 8 dpa a pygidium bearing three short cirri was restored (**d**–**f**, **j**–**l**). All pictures are in dorsal view. Scale bar 0.2 mm. White dashed lines show amputation region

We used a comparative transcriptomic approach in order to compare gene expression in three conditions: anterior regeneration (AR), posterior regeneration (PR), and non-regenerating (NR), i.e. intact individuals in regular posterior growth (see Figs. 1, 2 and 3 for experimental design and morphological data). mRNA samples of *S. hystrix* and *S. gracilis* were sequenced for each condition using an Illumina sequencing platform. Considering all three conditions, we generated a total of 79.5 GB raw reads for *S. hystrix* and 74.3 GB for *S. gracilis* (Table 1). After trimming the reads, 84.0 and 88.3% of reads remained for *S. hystrix* and *S. gracilis*, respectively (Table 1). Those cleaned reads were assembled, generating 315,224 contigs for *S. hystrix* (average length = 733.43, N50 = 1158) and 526,860 contigs for *S. gracilis* (average length = 626.48, N50 = 858). According to BUSCO [57], both transcriptomes were highly complete 97,8% (*S. hystrix*) and 98,6% (*S. gracilis*), despite showing a high level of redundancy with 73.8 and 80.6%, respectively (Table 1). We found 179,841 predicted proteins in the transcriptome of *S. hystrix* and 309,576 predicted proteins in the one of *S. gracilis* (Table 1). The raw reads were uploaded at the NCBI Sequence Read Archive (SRA). Assemblies and transdecoder predicted proteins are available under https://github.com/rannypribeiro/Regeneration_transcriptomics.

**Table 1:** Statistical summary of raw data, transcriptome assembly, and functional annotation of *Sphaerosyllis hystrix* and *Syllis gracilis*

Parameters	*Sphaerosyllis hystrix*	*Syllis gracilis*
Raw reads^a^	79.5 GB	74.3 GB
Total assembled bases	231,196,267	330,068,885
Total number of reads	122,278,261	113,602,020
Number of clean reads	102,763,252	100,322,750
Median contig length (nucleotides)	405	377
Average contig length (nucleotides)	733.43	626.48
N50 value (nucleotides)	1158	858
Total number of transcripts	315,224	526,860
Average lenght of transcripts (nucleotides)	642.64	546.32
Transcripts with GO annotation	90,058	128,997
Predicted proteins	125,040	184,632
Trinity ‘genes’	179,841	309,576
Completeness	97.8%	98.6%
Duplicated copies	73.8%	80.6%
Single copies	24.0%	18.0%
Fragmented copies	1.9%	1.3%

^a^Sum of raw reads of all sequenced libraries

### Functional annotation of transcripts and gene ontology

Around 35.7% (*S. hystrix*) and 31.3% (*S. gracilis*) of the assembled transcripts were annotated. The annotation results showed hits with human and mouse genes mostly, and less than 1% with known annelid genes (Additional file 1). Within Annelida, most transcripts were annotated with *Lumbricus* sequences: 38% (*S. hystrix*) and 28% (*S. gracilis*) (Additional file 1). Gene ontology (GO) categories were assigned to 28.5 and 24.5% of the transcripts of *S. hystrix* and *S. gracilis*, respectively. Our results showed that both species have a similar distribution of genes associated to the categories of cellular component, molecular function and biological process (Additional file 1).

### Comparison of gene expression profiles

In order to identify differentially expressed (DE) genes, we compared the transcriptomic profiles of anterior regeneration and posterior regeneration (AxP), anterior regeneration and non-regenerating condition (AxN), and posterior regeneration and non-regenerating (PxN) of both studied species.

#### *Sphaerosyllis hystrix*

**Fig. 4:**
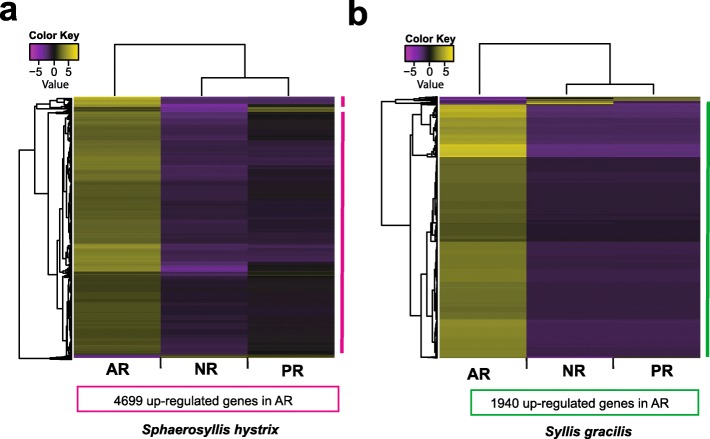
Heatmaps of differentially expressed genes during regeneration (FDR < 0.001). **a** *Sphaerosyllis hystrix* results. **b** *Syllis gracilis* results. Note that some of the genes can be up-regulated in more than one condition. Values in centred log_2_(fpkm+ 1). AR: anterior regeneration, PR: posterior regeneration, NR: non-regenerating. See Additional file 2: Table S1 and Additional file 3: Table S7 for detailed results

**Fig. 5:**
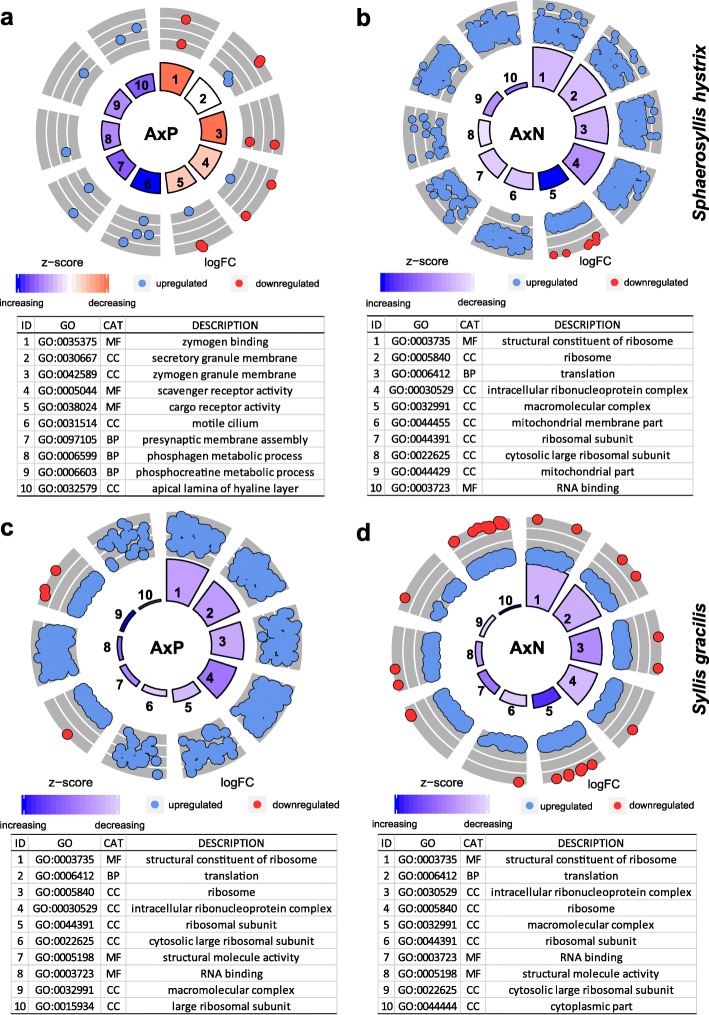
Results of gene ontology annotation of DE genes. Only the ten most significant enriched GO terms are plotted. **a** AxP comparison and **b** AxN comparison for *Sphaerosyllis hystrix*. **c** AxP comparison and **d** AxN comparison for *Syllis gracilis*. CAT: category; BP: biological process, CC: cellular component, MF: molecular function. Z-score is useful to know if the expression of genes belonging to a certain GO term is more likely to be decreasing (negative) or increasing (positive) and it is calculated as the number of up-regulated genes minus the number of down-regulated genes divided by the square root of the gene count [58]. Up-regulated genes have logFC> 0, and down-regulated genes have logFC< 0. Inner boxes size is based on the *p*-value and represents the significance of the enrichment of each GO term. Output data of the GOplot analyses is available in Additional file 2: Tables S5 and S6, and Additional file 3: Tables S11 and S12

Considering the overall results, we detected 4771 DE genes in *S. hystrix* (FDR < 0.001) (Fig. 4a; Additional file 2: Tables S1–S4). Analysing the comparisons separately, 108 genes were found to be differentially expressed in AxP, and 4768 genes in AxN. No DE genes were found in PxN. Four thousand six hundred sixty-three of the DE genes were exclusively found in AxN; 105 genes were present in both AxN and AxP; and only 3 genes were exclusive of AxP. Most of the DE genes were up-regulated in AR (4699) rather than in PR (161) or in NR (58) (Fig. 4a). AR up-regulated genes had similar expression levels in both PR and NR (see Additional files 2: Table S1). Gene Ontology analysis showed that 76% of the DE genes were annotated. The most prominent GO terms in AxP and AxN belong to the cellular component category (e.g. secretory granule, zymogen granule membrane, motile cilium, apical lamina of hyaline layer, ribosomal and mitochondrial parts) (Fig. 5a, b; Additional file 2: Tables S5 and S6).

#### *Syllis gracilis*

The overall results of the differential expression analysis showed 1997 DE genes among the three experimental conditions of *S. gracilis* (FDR < 0.001) (Fig. 4b; Additional file 3: Tables S7–S10). Of those genes, 1863 and 1428 were found in AxN and AxP, respectively. Similarly, to the results obtained for *S. hystrix*, no DE genes were found in PxN (FDR < 0.001). Of the DE genes, 529 were exclusive of AxN; 1334 were present simultaneously in AxN and AxP; and only 134 were exclusively detected in AxP. One thousand nine hundred forty genes were up-regulated in AR rather than in PR (33) or in NR (42) (Fig. 4b). In terms of gene ontology, 86% of genes with differential expression were annotated and the most prominent GO terms in AxP and AxN belong to the cellular component category (e.g., ribosome, intracellular ribonucleoprotein complex, ribosomal unit, macromolecular complex annotated) (Figs. 5c, d; Additional file 3: Tables S11 and S12).

### Identification of candidate regeneration genes

In order to identify putative regeneration-related genes in these species, BLAST searches were performed against our transcriptomes using publicly available sequences of those genes that have been previously shown to be (highly) expressed during regeneration in other annelids (Table 2; Additional file 4) [1, 2, 12, 13, 17, 21, 23, 27, 32, 35, 45, 46, 48, 59–63].

**Table 2:** Results of BLAST searches for candidate regeneration genes

	*Sphaerosyllis hystrix*			*Syllis gracilis*		
	Trinity gene ID	AxP	AxN	Trinity gene ID	AxP	AxN
β*-catenin*	TR65158|c1_g2	–	–	TR89060|c2_g1	+	+
*Brat*	TR63166|c4_g3	–	–	TR74232|c0_g1	+	+
*cycB3*	TR69437|c2_g1	–	–	TR101261|c2_g1	+	+
*Elav*	TR86647|c2_g1	–	–	TR79253|c1_g1	+	+
*FGFR*	TR95577|c0_g2	–	–	TR64245|c1_g1	+	+
*Gs*	TR72222|c3_g2	–	–	TR76174|c0_g1	+	+
*gcs1a*	TR38757|c0_g2	–	–	TR89735|c2_g1	+	+
*Hox7*	TR74688|c1_g5	–	–	TR50489|c0_g1	+	+
*JNK*	TR19529|c0_g1	–	+	TR151703|c0_g1	–	–
*Lox2*	TR72209|c0_g3	–	–	TR122252|c3_g1	+	+
*Paics*	TR25215|c0_g2	–	+	TR87989|c0_g1	+	+
*PL10*	TR66033|c1_g1	–	–	TR99989|c1_g1	–	+
*rup2*	TR83599|c2_g1	–	+	–	–	–
*Slit*	TR63077|c0_g1	–	–	TR107009|c0_g1	+	+

Plus signs indicate statistically significant differential expression results (FDR < 0.01). AxP: anterior regeneration versus posterior regeneration. AxN: anterior regeneration versus non-regenerating individual. NxP: non-regenerating individual versus posterior regeneration (see Additional files 2, 3 and 4 for complete results)

A total of 71 regeneration-related candidates were found in the literature. From those, 57 were identified in the transcriptome of *S. hystrix* and 54 in the transcriptome of *S. gracilis*. Multiple gene isoforms were identified after BLAST searches in *S. hystrix* (e.g. for *paics* and *slit*) and *S. gracilis* (e.g. *even-skipped*, *FGFR*, *gcs1a*, *glutamine synthetase*, *hedgehog*, *JNK*, *Msx*, *piwi1*, *Sfrp1/2/5* and *Wnt*) (Additional file 4), indicating that there might be multiple unique homologs of some of those genes in these species. Of the resulting homologs, *paics* in *S.hystrix*; and β*-catenin*, *cycB3*, *glutamine synthetase*, *paics*, and *PL10* in *S. gracilis* were detected to have differential expression, being all of them up-regulated in AR (FDR < 0.001). If we consider the significance threshold to be FDR < 0.01, the number of candidate regeneration genes with differential expression increases to 14, including *JNK* and *rup2*, in *S. hystrix*; and *brat*, *elav*, *FGFR*, *gcs1a*, *slit*, *Hox7*, *Lox2* in *S. gracilis* (Table 2; Additional file 4). Interestingly, all the *Hox* genes reported to be involved in the regeneration and development of other annelids [2, 3, 23, 46, 64] were found in the transcriptome of *S. hystrix* but none of them presented differential expression in any of the pairwise comparisons. In the case of *S. gracilis*, all *Hox* genes were found in the assembly, except *Hox2* and *Hox3*. Interestingly, *Hox7* and *Lox2* were among differentially expressed genes in the comparisons AxP and AxN, being up-regulated in AR (FDR > 0.01) (Table 2, Additional file 4).

### Morphological results of regeneration

The herein studied species exhibited a complete posterior regeneration, but anterior regeneration developed to different degrees. *Sphaerosyllis hystrix* regenerated an incomplete prostomium after 14 dpa and, even in advanced stages (around 50 dpa), they did not restore new segments. Thus, like in many other syllids [51, 65], the anterior regeneration of *S. hystrix* seems to be limited. Regarding *Syllis gracilis*, our own field observations and previous studies provide solid evidence that they are able to restore a complete anterior body with up to 18 segments and all digestive structures [37, 55, 66]. Moreover, specimens of *S. gracilis* from the same area showing advanced anterior regeneration have also been documented in detail by Parapar et al. [55]. *Syllis gracilis* was expected to regenerate the prostomium after 8 dpa, based on previous studies [37, 66]. However, we noticed only a blastema elongation during anterior regeneration after 8 dpa. This observed difference might be a result of the reduced temperature in our study (14 °C) compared to the one used by Boilly and Thibaut [37] (18 °C), as lower temperatures seem to delay the whole regeneration process in syllids [51].

## Discussion

### Posterior regeneration resembles regular posterior growth

In this study, we investigate regenerative processes of two species of syllids *Sphaerosyllis hystrix* (Exogoninae) and *Syllis gracilis* (Syllinae). Using comparative transcriptomics, we analyse three conditions: anterior regeneration, posterior regeneration, and regular growth. In both investigated species, our analyses revealed no differentially expressed (DE) genes between posterior regeneration (PR) and regular growth (NR); whereas the anterior regeneration (AR) significantly differed from those other conditions by having a high number of up-regulated genes. The absence of DE genes in the PxN comparisons of both species indicates that genes in PR and NR have similar expression levels. This result suggests that the genetic mechanisms behind the posterior regeneration and regular growth are similar in syllids with lifelong growth.

Previous studies provided similar results indicating that several genes expressed in the SAZ are also expressed in the blastema during posterior regeneration in annelids [3, 5, 21–23, 29, 46]. These two regions contain undifferentiated cells (blastema) and pluripotent cells (teloblasts in the SAZ), which require the activity of certain genes linked to stem-cellness, differentiation, reestablishment of antero-posterior and dorso-ventral axes, and elongation of the nervous system, among other processes [1, 3, 4, 7, 27, 46, 59]. Those processes are present during regeneration, growth, and homeostasis in planarians and acoels, and have been shown to be regulated by similar genetic pathways, e.g. Wnt and FGFRL signalling, TOR (target of rapamycin) control, and germline multipotency program activity [67–71]. Body growth and regeneration, therefore, are somehow similar programs in animals with high regenerative capacity.

### Gene up-regulation in the anterior regeneration

The high number of up-regulated genes in AR may be due to the combination of two different factors: First, the presence of two proliferative zones acting at the same time (the SAZ and the blastema of anterior regeneration (see Fig. 1). Second, as suggested by a previous study in flatworms [72], some DE genes in AR might be involved in the reestablishment of anterior identity and the regeneration of anterior-specific structures, such as the brain. The presence of two proliferative zones in AR implies the existence of a higher number of cells simultaneously expressing certain genes involved in regeneration and growth. Therefore, the overall number of reads of transcripts related to these processes might be higher in AR than in PR or NR. The GO annotation showed that most of the DE genes were assigned to the cellular component category, thus suggesting functions related to cellular proliferation.

However, many of the up-regulated genes in AR of both species could not be identified and, hence, their functions remain unknown. This is probably due to the scarcity of annelid genomic data in the databases used for annotation. In addition, the generated assemblies had high duplication levels, which resulted in artificially large transcriptomes. These high duplication levels were probably a consequence of pooling different individuals for each sequencing library, which can introduce allelic variation, splicing differences, and assembly artefacts [73]. Nevertheless, our transcriptomic analyses relied on highly complete assemblies based on BUSCO criteria and the comparison with other annelid assemblies [57, 74, 75]; and, since we performed the differential expression analyses at the level of Trinity ‘genes’ (which sums up the expression values of all isoforms of a ‘gene’), this redundancy does not affect our results.

### Regeneration-related genes

Using BLAST searches, we were able to identify regeneration-related candidates among the DE genes of our analysis. Those candidates have been associated to regenerative processes such as wound healing, blastema formation, stem cell regulation, cell proliferation, segmentation, and morphogenesis by several studies in annelids [1, 2, 12, 13, 17, 21, 23, 27, 32, 35, 45, 46, 48, 59–63]. Among the regeneration-related genes explored in this study, we found 12 DE genes in *S. gracilis* and 3 DE genes in *S. hystrix*; all of them were up-regulated in AR (FDR > 0.01, see Table 2).

Some of the DE genes are associated to cell proliferation and nervous system elongation, processes that support the two-proliferation-zones hypothesis suggested above; they are *paics, JNK*, *PL10*, *slit*, *elav*, glutamine synthetase (*gs*), and *rup* [29, 46, 76, 77]. Of these candidate genes, only *paics* (phosphoribosylaminoimidazole) was differentially expressed in both species (Table 2). *paics* is required for de novo biosynthesis of purines during cellular proliferation, and it has been reported to be highly expressed during regeneration in the clitellate *Enchytraeus japonensis* [12]. Similarly, *JNK* (up-regulated homolog in AR of *S. hystrix*) translates signals into apoptotic cell death and controls cell proliferation and differentiation to coordinate regeneration in planarians [78, 79]. Also, a homolog of *PL10* was differentially expressed in *S. gracilis* results, up-regulated in AR (AxN comparison). *PL10,* like *vasa*, *piwi*, and *nanos*, is one of the germline multipotency program genes [80]. These genes are linked to somatic differentiation and stem-cellness, and can be considered conserved markers of the SAZ in annelids [14, 26, 34, 48, 62, 74, 80, 81].

The genes *gs*, *elav* and *slit* play an important role in nervous system regeneration and growth in annelids [12, 13, 46]. Homologs of those genes were found to be up-regulated in AR (AxP and AxN comparisons of *S. gracilis*). The enzyme glutamine synthetase (encoded by *gs*) plays a role in cell metabolism, ammonia detoxification, glutamate transmitter degradation in the nervous system, and was found to be expressed in early stages of regeneration in *Enchytraeus japonensis* [12, 13, 82]. The genes *slit* and *elav*, on the other hand, encode signalling and a RNA-binding proteins, respectively [83, 84]*.* They are expressed, for example, in the ventral midline cells (*slit*) and differentiating neurons (*elav*) during posterior regeneration in *Platynereis dumerilii* [46], and are evolutionary conserved across animal evolution [83, 84].

Interestingly, in both transcriptomes we identified homologs of *Ej-rup 1–5* (*E. japonensis* regeneration up-regulated genes 1–5), regeneration-related genes previously reported for the clitellate *E. japonensis* (Table 2) [12]. However, only in *S. hystrix* one of them (*Shy-rup2*) was up-regulated in AR (AxN comparison). The function of this gene is not clear, but *Ejrup2* was detected in epidermal cells of the blastema during anterior regeneration and might be a regeneration-specific gene [12].

Supporting the hypothesis of AR gene up-regulation being related to the reestablishment of anterior identity and structures, we detected some DE genes probably related with the specification of the antero-posterior axis, e.g. *Hox* genes and β-*catenin* [21–23, 64, 69, 85, 86]. In this study, *Sgr-Hox7* and *Sgr-Lox2* were up-regulated in AR (AxP and AxN comparisons; FDR > 0.01), which means that they are expressed in similar levels during tail regeneration and regular posterior growth, but are required during anterior regeneration of *S. gracilis*. In studies on the annelids *P. dumerilii* and *A. virens* (which cannot regenerate anteriorly), the expression of *Hox7* and *Lox2* was detected during larval development, growth and posterior regeneration [21–23, 64]. In addition, we detected up-regulation of a homolog of β-*catenin* in AR of *S. gracilis* (AxP, AxN comparisons; FDR < 0.001). In annelids, for example, β-*catenin* expression has been found in the blastema of *P. leidyi* during anterior and posterior regeneration, and in fission zones during asexual reproduction [17]. Additionally, Demilly et al. [59] suggested that the *Wnt*/β-*catenin* pathway is involved in neural cell proliferation/differentiation in *P. dumerilii*. In planarians, *Wnt*/β-*catenin* signalling is known to be required for the establishment of the antero-posterior axis during regeneration, promoting homeostasis and proper brain regeneration [67, 69, 85].

## Conclusions

We studied the regenerative abilities of two syllid species. Both species can completely regenerate the posterior body after one to 2 weeks post amputation. However, only *Syllis gracilis* is able to regenerate the entire anterior body and, in contrast, *Sphaerosyllis hystrix* has a limited anterior regeneration. By using RNA-seq, we found that, for both species, individuals in posterior regeneration and intact individuals have comparable gene expression profiles. On the other hand, anterior regeneration shows a significant up-regulation of DE genes, including some candidate regeneration genes related to cellular proliferation (*paics* and *JNK*), nervous system development (*gs*, *elav*, *slit*), stem-cellness (PL10), and reestablishment of antero-posterior axis (*Hox* genes and β*-catenin*). Those results lead to two main conclusions, first that posterior regeneration is similar to the postembryonic process of growth in annelids, while anterior regeneration is markedly different from both; and second, that syllids regenerate using common genetic pathways (regeneration-related genes) already described for other annelids and other groups of invertebrates, supporting the importance of comparative studies to illuminate the evolution of regeneration in Metazoa.

## Methods

### Sampling

Animals were collected in intertidal rocky shores from Ferrol, Galicia, Spain (43°27′17.0″N; 8°18′39.8″W) during third quarter moon in April 2017. Specimens of *Syllis gracilis* and *Sphaerosyllis hystrix* were sorted for regeneration experiments. *Syllis gracilis* is a species complex with eight recognized lineages, from which a specimen from Galicia, Spain has been shown to belong to ‘lineage 8′ [87]. We confirmed that our specimens also belong to this lineage through phylogenetic analysis using sequences from the transcriptome assembly and those provided by a previous study [87] (see Additional files 5 and 6). For each molecular marker (COI, 16S, 18S, 28S), alignments were performed using MAFFT version 7 [88] (G-INS-I iterative method), and the datasets produced were concatenated using FASconCAT-G version 1.02 [89]. Then, a maximum likelihood analysis was conducted using RAxML, with 1000 bootstrap pseudoreplicates, and a partition scheme allowing for optimization of the three genes separately [90, 91].

### Experimental procedures

Intact non-reproducing adults were selected for regeneration experiments. The animals were anesthetized in a 3.5% MgCl_2_ solution dissolved in seawater. Bisection was performed in the midbody of 48 individuals of *S. hystrix* (after chaetigers 13–18), and of 30 individuals of *Syllis gracilis* (after chaetigers 25–48) (Fig. 1). The animals were kept in one-litre aquariums with flowing filtered natural seawater at 14 °C for up to 14 dpa. Since there were only few specimens of *S. gracilis*, and some of them died during experimentation, the regeneration process could only be followed until 8 dpa. Anterior and posterior amputees were separated in different aquaria and kept in starvation during the experiment. Two amputees of each condition were fixed in 4% PFA every day for morphological observations (up to 14 dpa in *S. hystrix* and 8 dpa in *S. gracilis*, see Fig. 1). Optical microscopy images of fixed animals were taken to document morphological changes using an Olympus CX31 microscopy and a BQ Aquaris V. For transcriptome sequencing, amputees were fixed in RNA later (Ambion, Darmstadt, Germany) to represent four stages of regeneration: stage 1 (healing response/cicatrisation); stage 2 (early blastema development); stage 3 (late blastema development); stage 4 (patterning/cap regeneration), only observed during posterior regeneration (see Fig. 1). Two amputees of *S. hystrix* were fixed per stage: 1 dpa (stage 1), 5 dpa (stage 2), 9 dpa (stage 3) and 12 dpa (stage 3/ stage 4), summing up a total of 16 amputees; i.e. 8 for anterior regeneration (AR) and 8 for posterior regeneration (PR). Additionally, five intact individuals of *S. hystrix* were fixed as non-regenerating condition (NR). For *S. gracilis,* one amputee of each regenerative condition (AR, PR) was fixed in 1 dpa (stage 1), 3 dpa (stage 2), 6 dpa (stage 3) and 8 dpa (stage 3/stage 4), and two whole animals were fixed for NR (Fig. 1). All experimental procedures were conducted in April to May 2017 at the Marine Biological Station of A Graña (Ferrol, Galicia, Spain).

### Illumina sequencing and de novo assembly

Three libraries were prepared for each of the two species: anterior regeneration (AR), posterior regeneration (PR), and non-regenerating adults (NR), i.e. intact individuals in regular posterior growth. RNA extraction was conducted by pooling together all individuals belonging to the same condition and species, i.e. amputees in different stages were pooled for each regenerative condition (AR, PR) and intact specimens were pooled together to prepare the non-regenerating condition (NR). Considering that the sequences represent samples of pooled individuals, there were no biological replicates from which to estimate inter-individual variability in gene expression. Although replicates provide a robust statistical support in differential expression analyses [92, 93], pooling samples for transcriptomic sequencing can be a useful strategy to establish a good framework of DE genes from small animals and neglected organisms [94]. RNA was isolated using NZYTech’s Total RNA isolation kit and the pure RNA was eluted in a final volume of 30 μL. Quality and quantity of RNA were checked in an Agilent 2100 Bioanalyzer using Agilent RNA 6000 kit. To prepare the libraries, we used Illumina’s TruSeq Stranded mRNA Library Prep Kit following manufacturer’s instructions. Fragment size distribution and concentration were checked in the Agilent Bioanalyser. Qubit dsDNA BR Assay kit (Thermo Fisher Scientific) was used to quantify the libraries, which were then sequenced in an Illumina HiSeq 4000 PE100 lane. All procedures of RNA isolation, library construction, and sequencing were performed by AllGenetics & Biology SL (A Coruña, Spain).

We used FastQC v0.11 (http://bioinformatics.babraham.ac.uk/projects/fastqc/) to assess sequence quality, and Trimmomatic v0.33 [95] to trim the raw sequences based on quality results with options HEADCROP:10 LEADING:20 SLIDINGWINDOW:5:20 MINLEN:70. De novo transcriptome assembly was perform using Trinity v2.3.2 [96, 97] and transcripts with ≥200 bases were kept. Assembly statistics were obtained using the TrinityStats tool of Trinity, and BUSCO [57] was used to estimate transcriptome completeness.

### Functional annotation and gene ontology

Transcripts were annotated following the Trinotate pipeline (https://github.com/Trinotate/Trinotate.github.io/wiki). For that, TransDecoder v3.0.1 (https://transdecoder.github.io/) was used to predict protein sequences from the assembled transcripts. Then, both the assembled transcripts and the predicted proteins were used for functional annotation, which was performed using BLAST v2.5.0 [98], HMMER v3.1b2 (http://hmmer.org), signalp v4.1 [99], RNAmmer v1.2 [100], and tmHMM v2.0c [101] to find known sequences and domains. Gene ontology (GO) terms analysis was used to classify the functions of the predicted genes. Trinotate v3.0.1 (http://trinotate.github.io) and WEGO Web Server [102] were used to summarise the results of gene ontology (GO).

### Differential expression analyses

Using the Differential Expression module of Trinity v.2.3.2.

(https://github.com/trinityrnaseq/trinityrnaseq/wiki/Trinity-Differential-Expression), we performed pairwise comparisons at the Trinity ‘gene’ level between each condition of our experiment: anterior regeneration against posterior regeneration (AxP), anterior regeneration against non-regenerating condition (AxN), and posterior regeneration against non-regenerating (PxN). We ran RSEM [103] to estimate per-condition transcript abundance, and edgeR [104, 105] to perform the differential expression analyses. At this point, we tried several values for the *dispersion* parameter of edgeR, and concluded that 0.5 was the best fitting value for our data.

After that, we used the analyze_diff_expr.pl script of Trinity to create differential expression subsets for each pairwise comparison, and included the *examine_GO_enrichment* flag that, by combining the results of differential expression and the functional annotation, can inform which Gene Ontology categories are enriched or depleted in each experimental condition for each pairwise comparison. We used GOplot to illustrate the results of gene ontology (GO) enrichment analyses [58]. The outputs files of the differential expression analyses and the functional enrichment analyses are available under https://github.com/rannypribeiro/Regeneration_transcriptomics. Overall results are summarised in Additional files 2 and 3.

### Identification of candidate regeneration genes

BLASTn and BLASTp searches were used to detect homologs of genes related to animal regeneration that had been previously reported in the literature. The top hits in the BLAST results were analysed via a reciprocal BLASTn against the nr/nt database (NCBI) to verify the putative identity of candidate regeneration genes in the transcriptomes of *S. hystrix* and *S. gracilis*.

## Supplementary information

**Additional file 1. **Results of functional annotation of the transcriptomes of *Sphaerosyllis hystrix* and *Syllis gracilis*. **a** Results against all metazoan database. **b** Results within Annelida. **c** Gene ontology distribution of the annotated genes grouped in the three main functional categories (cellular component, molecular function, and biological process). GO terms with percentage of genes > 4% were plotted.

**Additional file 2. **Differential gene expression and functional enrichment results for *Sphaerosyllis hystrix*.

**Additional file 3. **Differential gene expression and functional enrichment results for *Syllis gracilis*.

**Additional file 4.** BLAST results of candidate genes including differential gene expression results. ns: not significant.

**Additional file 5. ***Syllis gracilis* phylogeny including sequences identified from the transcriptome assembly of this study. The lineages are in agreement with previous study [87].

**Additional file 6. **GenBank accession numbers of sequences used for phylogenetic reconstruction. Codes used for *S. gracilis* sequences by Alvarez-Campos et al. [87] were maintained here.

## Data Availability

The raw reads are available at the NCBI Sequence Read Archive (SRA) for *Sphaerosyllis hystrix* (BioProject ID PRJNA517681, SRX5314036–38) and *Syllis gracilis* (BioProject ID PRJNA517687, SRX5314271–73). Assemblies, transdecoder predicted proteins, and differential gene expression results generated in this study can be accessed in https://github.com/rannypribeiro/Regeneration_transcriptomics.

## Abbreviations

AR: Anterior regeneration, as experimental condition
AxN: Anterior regeneration versus non-regenerating
AxP: Anterior regeneration versus posterior regeneration
BP: Biological process
BUSCO: Benchmarking Universal Single-Copy Orthologs
CC: Cellular component
DE genes: Differentially expressed genes
dpa: Day(s) post amputation
*Ej*-rup: *Enchytraueus japonensis* regeneration up-regulated gene
FGFR: Fibroblast growth factor receptor
Gcs1a: Glucosidase 1
GO: Gene ontology
gs: Glutamine synthetase
GSK3β: Glycogen synthase kinase-3 β
JNK: c-Jun N-terminal kinase
MF: Molecular function
NR: Non-regenerating, as experimental condition
paics: Multifunctional protein ADE2
PR: Posterior regeneration, as experimental condition
PxN: Posterior regeneration versus non-regenerating
