# Supplementary figures and images for: Comparative transcriptomics in Syllidae (Annelida) indicates that posterior regeneration and regular growth are comparable, while anterior regeneration is a distinct process
Source: BMC Genomics. 2019 Nov 14;20:855. doi: 10.1186/s12864-019-6223-y (PMC6854643; doi:10.1186/s12864-019-6223-y)

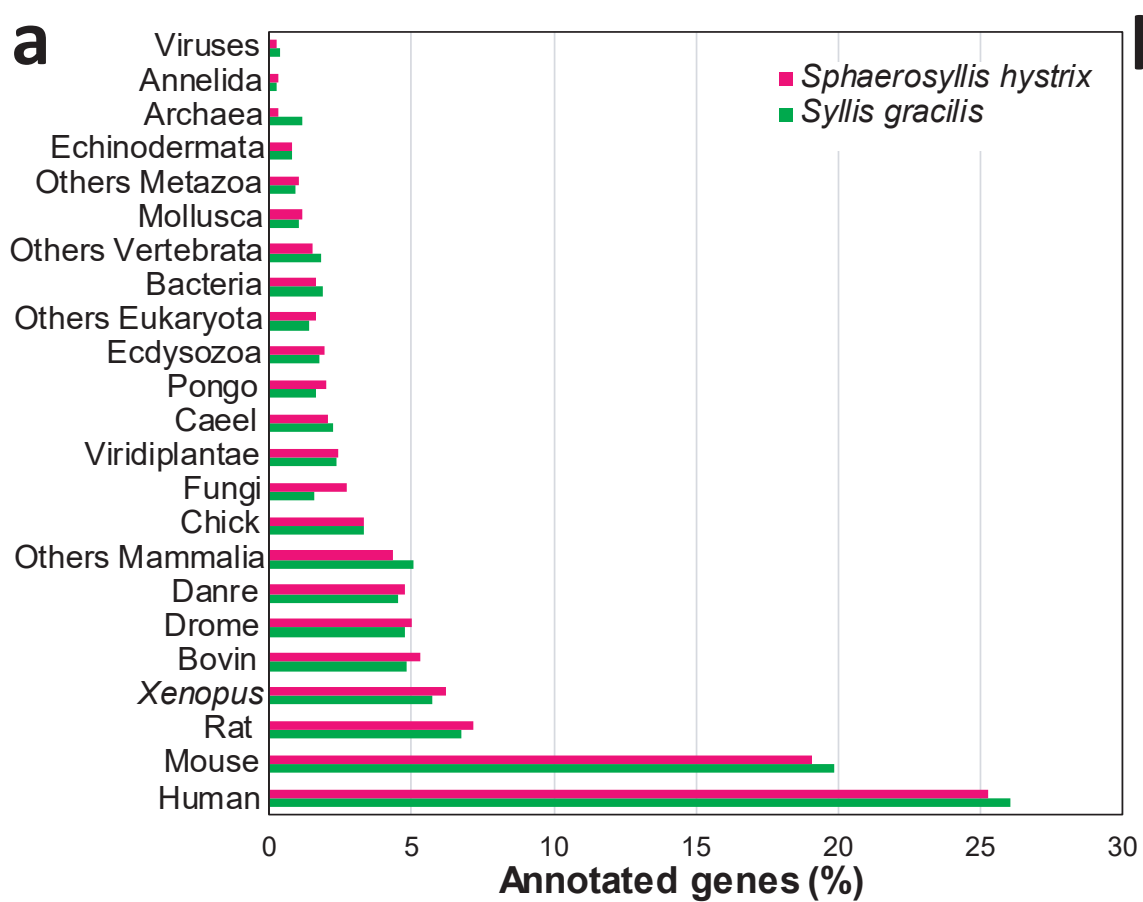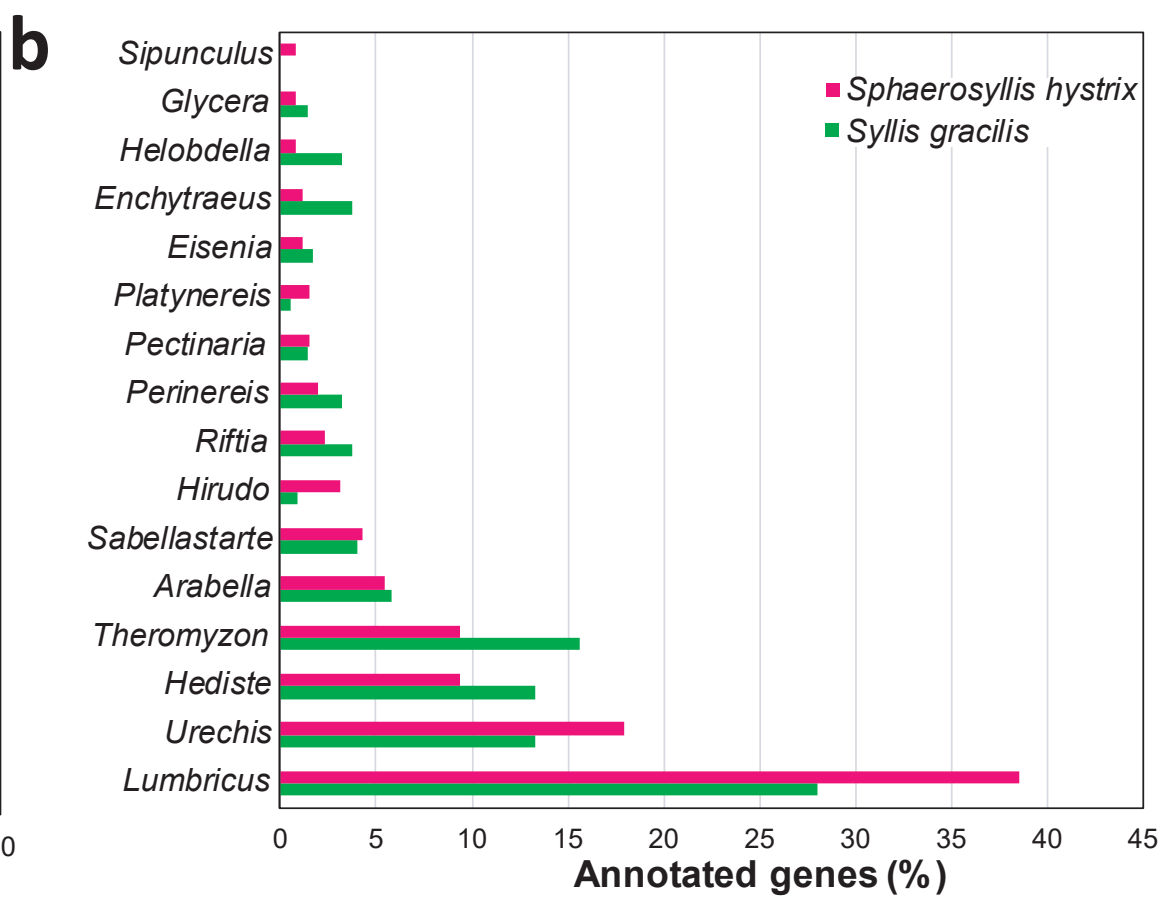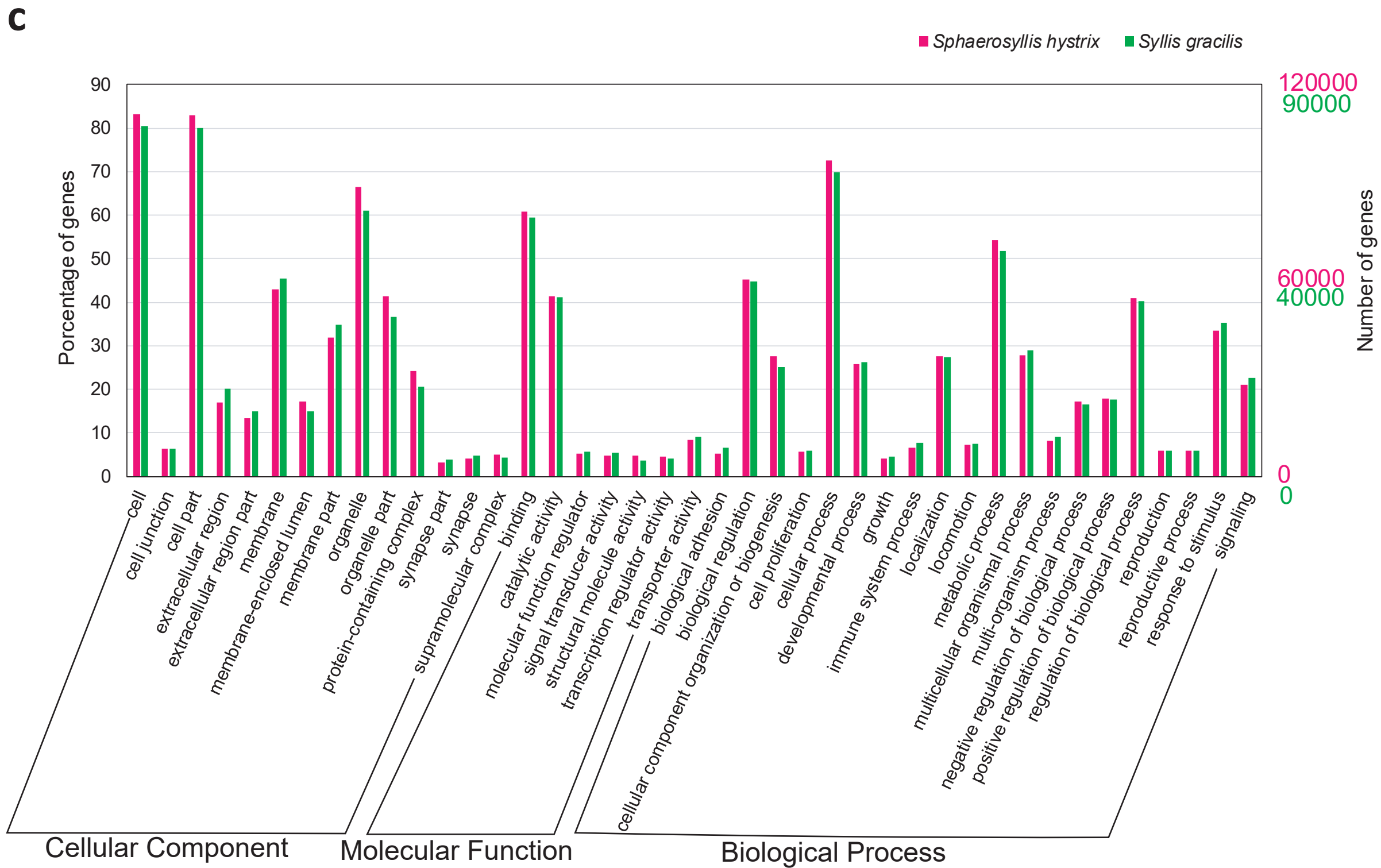

Supplement: Supplementary file 1 — Additional file 1. Results of functional annotation of the transcriptomes of Sphaerosyllis hystrix and Syllis gracilis. a Results against all metazoan database. b Results within Annelida. c Gene ontology distribution of the annotated genes grouped in the three main functional categories (cellular component, molecular function, and biological process). GO terms with percentage of genes > 4% were plotted. [file 12864_2019_6223_MOESM1_ESM.pdf]

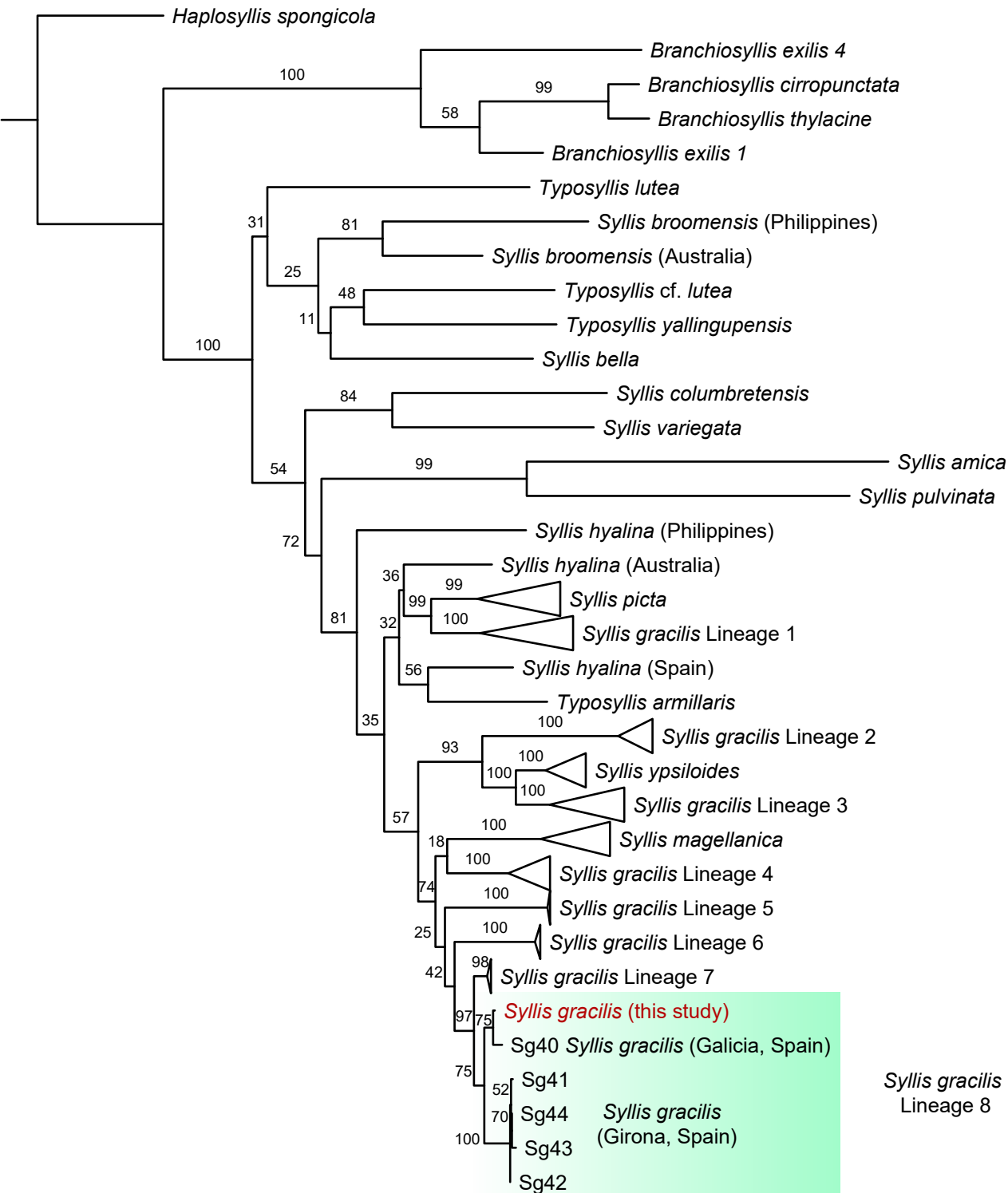

Supplement: Supplementary file 5 — Additional file 5. Syllis gracilis phylogeny including sequences identified from the transcriptome assembly of this study. The lineages are in agreement with previous study [87]. [file 12864_2019_6223_MOESM5_ESM.pdf]
